# Kinetic studies of inverse electron demand Diels–Alder reactions (iEDDA) of norbornenes and 3,6-dipyridin-2-yl-1,2,4,5-tetrazine

**DOI:** 10.1016/j.tetlet.2014.07.002

**Published:** 2014-08-20

**Authors:** Astrid-Caroline Knall, Manuel Hollauf, Christian Slugovc

**Affiliations:** Institute for Chemistry and Technology of Materials (ICTM), Graz University of Technology, Stremayrgasse 9, A-8010 Graz, Austria

**Keywords:** Tetrazines, Norbornenes, Click chemistry, Kinetics, Inverse electron demand Diels–Alder reactions

## Abstract

Inverse electron demand Diels–Alder additions (iEDDA) between 1,2,4,5-tetrazines and olefins have recently found widespread application as a novel ‘click chemistry’ scheme and as a mild technique for the modification of materials. Norbornenes are, due to their straightforward synthetic availability, especially interesting in the latter context. Therefore, the reactivity of different norbornene-based compounds was compared with unsubstituted norbornene and other alkenes using UV-vis measurements for the determination of reaction rates under pseudo first order conditions. Thereby, *exo*,*exo*-5-norbornene-2,3-dimethanol was found to be almost as reactive as unsubstituted norbornene whereas (±)-*endo*,*exo*-dimethyl-5-norbornene-2,3-dicarboxylate reacted only insignificanty faster than unstrained alkenes.

## Graphical abstract

**Figure:**
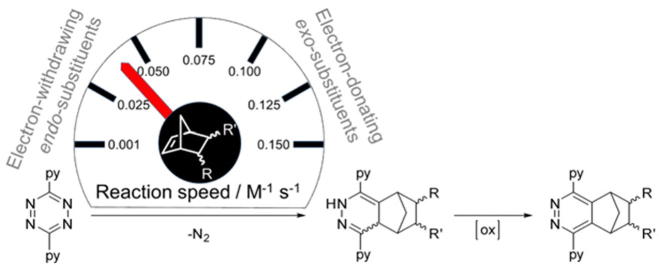

## Introduction

**Scheme 1:**
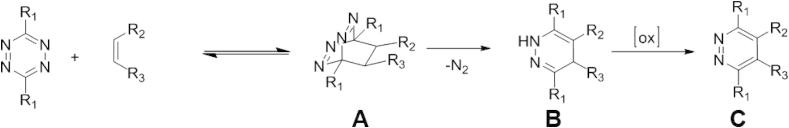
Inverse electron demand Diels–Alder (iEDDA) reaction leading to the formation of pyridazines.

Inverse electron demand Diels–Alder additions (iEDDA) are known to take place between 1,2,4,5-tetrazines and olefins. Thereby, a bicyclic intermediate **A** is formed which readily eliminates nitrogen and rearranges resulting in dihydropyridazines (**B**), which may be further oxidized to pyridazines (**C**, Scheme 1). In contrast to Diels–Alder reactions with ‘normal’ electron demand, the HOMO of the dienophile and the LUMO of the diene interact in iEDDA reactions.^1^ Therefore, while electron-deficient tetrazines react faster due to a lowered LUMO, the olefinic dienophiles should ideally be electron-rich. Additionally, substituents which are sterically hindering on either reaction partner were found to reduce the reaction rate. Furthermore, strained double bonds are known to react faster in iEDDA reactions.^2^ While these general rules apply for both alkenes and alkynes, alkynes were found to generally react slower in iEDDA reactions, but directly lead to pyridazines as reaction products.

Previously, iEDDA reactions have been predominantly used for the preparation of pyridazines, which were applied as nitrogen donor ligands.^3^ In 2008, however, two groups independently discovered that the fast and selective iEDDA reaction of strained olefins and tetrazines can be used in the sense of ‘click chemistry’ fulfilling most of Sharpless’ criteria.^4,5^ Moreover, no catalyst or excess reagent needs to be applied which has to be separated off after the conjugation reaction. This, together with high functional group tolerance and high reaction rates has led to numerous applications of iEDDA reactions in life science as a bioorthogonal conjugation scheme, meaning that the biological activity of biological entities such as enzymes and even whole cells is not disturbed. In molecular biology, high reaction speed and, moreover, hydrolytic stability are key for successful click reactions^6^ which is achieved by combining comparably unreactive, but hydrolytically stable tetrazines with highly reactive olefinic conjugation partners, such as *trans*-cyclooctene.

Since both reaction partners can be selected from a wide variety of compounds, however, the speed of iEDDA reactions can be tuned by selecting appropriate dienes and dienophiles. Thus, using olefins with less ring strain or a higher degree of substitution in iEDDA reactions becomes possible, if more reactive tetrazines are used (i.e., less sterically hindered or more electron-deficient) or elevated temperatures are applied. The hydrolytic stability of the tetrazine reaction partners is also less critical for applications in materials science (since aqueous reaction conditions are not mandatory) thus enabling the use of more reactive tetrazine species. Furthermore, the reaction temperature is not limited to physiologically relevant conditions thus enabling the use of less reactive dienophiles. Very recently, iEDDA has therefore also raised a high amount of interest in materials science as a potential conjugation technique and a facile method for material modification.^7^ Recent examples include the preparation of block copolymers^8,9^ and side chain modification of biodegradable lactide polymers^10^ as well as amphiphilic block copolymers.^11^ Molecules bearing two or more tetrazine residues have been applied for crosslinking leading to single-chain polymer nanoparticles^12^ or hydrogels.^13,14^ Furthermore, tetrazines have been used to modify MOFs (metal-organic frameworks),^15^ fullerenes (which then were used as modifier layers in solar cells),^16^ carbon nanotubes^17,18^ and macroporous polymer foams.^19^ Due to their straightforward synthetic availability, norbornenes have been more abundantly used in materials science than *trans*-cyclooctenes (which are also known to readily isomerize into the unreactive *cis*-form). Therefore, while several previous studies have dealt with determining the reaction rates of different tetrazine-olefin iEDDA reactions,^20–22^ especially on substrates relevant for applications in molecular biology, we became interested in the reaction rates of norbornene derivatives in order to evaluate their reactivities compared to other alkenes which might be useful in the field of material modification by tetrazine-olefin additions.

## Experimental

Our intention was to investigate the reactivity of building blocks which could be of use in materials science, especially for the synthesis of macromolecules. 5-Norbornene-2-methanol has been identified to be one of the most reactive norbornenes in iEDDA reactions^20^ and was also used previously as a conjugation handle.^8^ Our aim and choice of substrates mainly were disubstituted norbornenes since they can be conveniently converted into alternating copolymers or allow assembling more complex entities,^23^ such as miktoarm star polymers. These were compared with unsubstituted norbornene (**1**), 5-norbornene-2-methanol (**2**) and other commercially available alkenes (**14**–**17**). **pyTz** (3,6-dipyridin-2-yl-1,2,4,5-tetrazine) was used in most of the preceding studies on material modification and was therefore selected to compare the reactivities of the different norbornenes mentioned above.

Norbornene-based substrates were obtained from (hetero) Diels–Alder reactions of either freshly distilled cyclopentadiene (**10**, **11**) or furan (**8**, **12**), respectively, with maleic anhydride or maleimide. For example, *endo*-5-norbornene-2,3-dicarboxylic acid anhydride (**10**) was prepared from maleic anhydride and cyclopentadiene^24^ whereas when furan was used as diene, the 7-oxa analogue **8** was obtained in *exo*-configuration.^25^ *N*-Benzyl-2-azanorbornene (**13**) was prepared from a Mannich dienophile generated in situ from benzylamine hydrochloride and formaldehyde.^26^ *Exo*-5-norbornene-2,3-dicarboxylic acid anhydride (**7**) was prepared by thermal isomerization of **10**.^27^ For the differently substituted norbornene alcohols **3**, **4** and **5**, the corresponding acid anhydrides, esters, (or, in the case of amino alcohol **6**, the Diels–Alder reaction product of maleamic acid and cyclopentadiene) were reduced using lithium aluminium hydride.^28^ **pyTz** was prepared according to a Pinner-type synthesis described in the literature.^19,29^ Substrates **1** (norbornene), **2** (5-norbornene-2-methanol), **9** ((±)-*endo*,*exo*-dimethyl-5-norbornene-2,3-dicarboxylate), **14** (dicyclopentadiene), **15** (cyclopentene), **16** (1-hexene) and **17** (styrene) were obtained from commercial sources (Sigma–Aldrich, Fluka, ABCR, Alfa Aesar) and used without further purification.

Generally speaking, the first reaction step, which is the Diels–Alder reaction with inverse electron demand, can be regarded as the rate-determining step in the whole reaction cascade, as confirmed by DFT calculations.^30^ Therefore, the reaction rates of iEDDA reactions can be straightforwardly determined by monitoring the decay of tetrazine concentration in the presence of excess dienophiles (pseudo first order conditions).^6,20–22,30^

**Figure 1:**
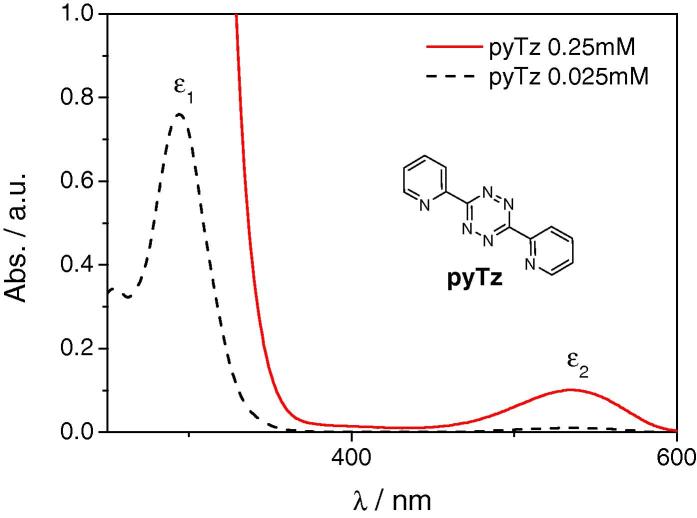
UV-vis absorption spectrum of 3,6-dipyridin-2-yl-1,2,4,5-tetrazine (**pyTz**).

The UV-vis absorption spectrum of **pyTz** shows two characteristic maxima, a stronger one at around 350 nm [*ε*_1_ = 30400 L mol^−1^ cm^−1^] and a weaker absorption maximum at around 550 nm [*ε*_2_ = 400 L mol^−1^ cm^−1^], which is responsible for the pink colour of the compound (Fig. 1). Since we observed that some of the formed pyridazines appeared yellowish, we decided to use the weaker absorption maximum to avoid interference with potentially absorbing products.

**Figure 2:**
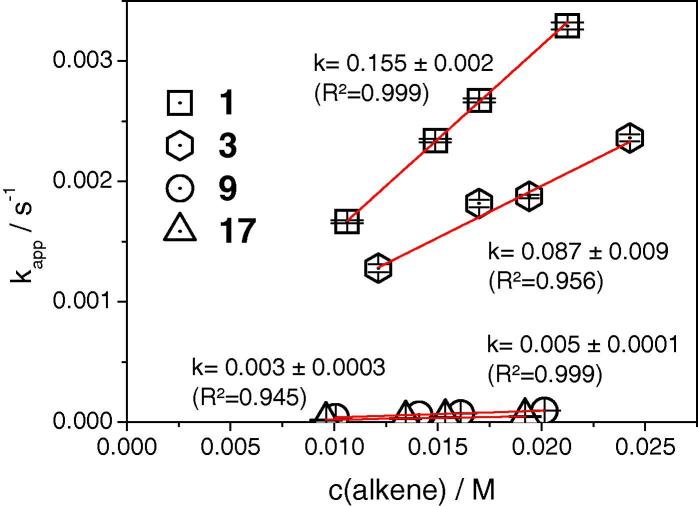
Linear dependence of pseudo first order reaction rate constants on the alkene concentration for **1**, **3**, **9** and **17**.

For the kinetic experiments, solutions of **pyTz** and the respective alkene (both in methanol) were mixed in cuvettes in a UV-vis photometer so that a final concentration of 1 mM **pyTz** and 10, 14, 16 and 20 mM of alkene substrate was achieved and the iEDDA reaction was initiated. Immediately after, the decay of tetrazine concentration was monitored for five minutes (10 s interval) at a wavelength of 545 nm. The pseudo-first order reaction rate constants were then determined by linear fits of ln([pyTz]/[pyTz_0_]) plotted versus reaction time. This procedure was repeated three times for each concentration. Linear regression with the alkene concentration allowed deriving the second rate order constants for the respective alkene–tetrazine couples (shown in Fig. 2).

## Results and discussion

Table 1 shows the determined second order reaction rate constants for all substrates. In previous publications, overall higher reaction rates for comparable norbornene–tetrazine couples have been observed^20,22^ which has the reason in the fact that methanol/water mixtures were used while we determined the reaction rates using pure methanol as solvent. Water is known to accelerate Diels–Alder reactions, which has been shown^31^ for iEDDA reactions of styrene and **pyTz** and later, also for norbornene–tetrazine reactions.^22^ The effect of *exo*-substituted norbornenes reacting significantly faster than *endo*-substituted norbornenes^20^ is even more pronounced in disubstituted derivatives leading to a decent reaction rate of *exo*,*exo*-5-norbornene-2,3-dimethanol (**3**) which was only 40% lower than in the case of unsubstituted norbornene. While *exo*-addition seems to be energetically favoured,^20^ a mixture of dihydropyridazines resulting from *exo*- and *endo*-attack on the norbornene is typically obtained, which could explain these observed differences in reactivity.^5,20^ In the case of **2**, a commercially available mixture of *endo*- and *exo*-5-norbornene-2-methanol (mainly containing the *endo*-derivative) was used. It should be noted here that according to the work of Carell et al. the reaction rate constant of the pure *exo* compound was almost 3 times higher than the pure *endo* compound.^20^

Unlike for cyclooctenes^32^ and cyclooctynes,^30^ it has been shown that the reactivity of *exo*-substituted norbornenes could not be further increased by incorporation of a cyclopropane ring. **6**, in which one of the CH_2_OH groups in 2- and 3-position was replaced by a CH_2_NH_2_ group (both in *endo*-configuration), was found to react twice as fast as **5**. This, together with a comparably higher reaction rate of **13** versus **2** and **12** versus **8** suggests a contribution of the nitrogen lone pairs, for example by pre-coordination of the tetrazines. Indeed, interactions between tetrazines and amines have been observed and utilized in fluorescence quenching experiments.^33^ Norbornenes **7**, **9** and **10** showed the same tendency with respect to stereochemistry while the electron-withdrawing substituents had a strong overall rate reducing effect (about one order of magnitude slower). In addition to this minus-I effect, also increased steric hindrance should be considered which will be more pronounced in the case of the carboxylic esters and anhydrides compared to substrates with CH_2_OH groups. **9** serves as a model compound for norbornenedicarboxylic diesters, which are often used as monomers in ring-opening metathesis polymerization because of their favorable reaction kinetics. Furthermore, such compounds are easily accessible by Diels–Alder addition of cyclopentadiene and fumaroyl chloride followed by esterification. Unfortunately, the reactivity of **9** was found to be only slightly higher than the reaction rate for cyclopentene (**15**), hexene (**16**) or styrene (**17**).

Previously, a rate-reducing effect of heteroatoms was found for 7-oxa norbornenes versus their homocyclic analogues.^20^ In our measurements, the reaction rates for **7** and **8** were found to be very similar whereas those of **11** and **12** were different by a factor of 20, which again highlights the role of steric effects. For *N*-benzyl-2-azanorbornene (**13**), a lower reaction rate compared to **2** was found which can be explained by increased steric shielding of the double bond by the aromatic ring. The reaction rate for unsubstituted norbornene compared to cyclopentene (**15**) is about one order of magnitude higher (caused by the difference in ring strain). Similarly, unstrained alkenes such as hexene (**16**) or styrene (**17**) reacted even more slowly.

In our previous paper on iEDDA grafting of dipyridyl(pyridazines) to an emulsion-templated macroporous polydicyclopentadiene (pDCPD) foam,^19^ we also performed studies on short linear pDCPD chains. Therein, no pronounced preference for certain double bonds within the pDCPD network was found which, considering the fact that **15** reacted almost 10 times faster than hexene (**16**), is most likely attributed to steric hindrance. The corresponding monomer, dicyclopentadiene (DCPD, **14**) itself is a highly interesting substrate because its two double bonds should possess different reactivities in iEDDA reactions due to their different amount of ring strain which is obvious comparing the reaction rates of **15** and **1**. The reaction rate of **14** was overall lowered compared to **1** by about 30%. To confirm that the preferred site for an iEDDA reaction is the more strained norbornene double bond, 1 equiv of **pyTz** and **14** each was mixed in a scintillation vial in dry dichloromethane and stirred for 24 h. Then, this product mixture was stirred with 1 equiv of DDQ in dichloromethane overnight and purified by silica gel filtration.

**Figure 3:**
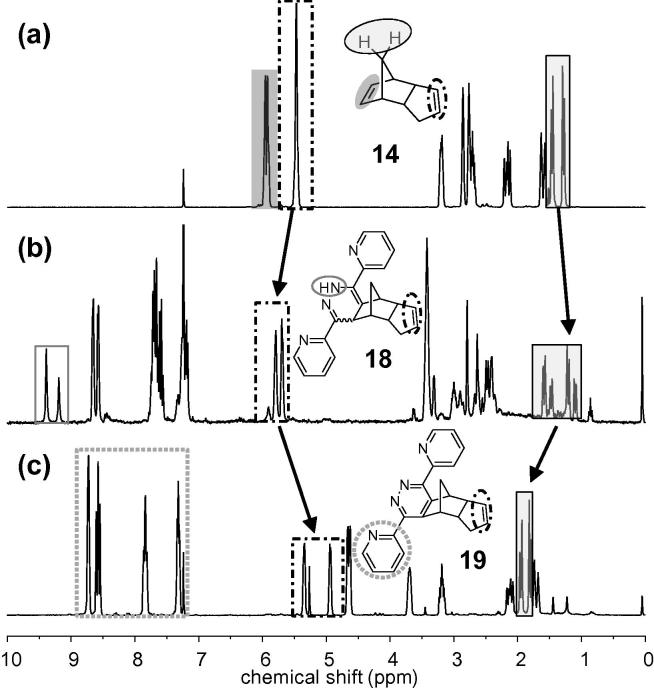
^1^H NMR spectra of (a) **14**, (b) after addition of **pyTz**, (c) after oxidation with DDQ (2,3-dichloro-5,6-dicyano-1,4-benzoquinone).

The ^1^H NMR spectrum of **14** (Fig. 3a) shows two olefinic double bonds, one at 5.2 ppm and one at 6 ppm. While the latter is consumed in the iEDDA reaction, the former notices a strong upfield shift (to approx. 5.8 ppm). Notably, the two adducts **18a**/**b** (Fig. 3b) show significantly different chemical shifts which is most obvious for the NH signal of the dihydropyridazines (at 9.2 and 9.4 ppm) and the norbornene CH_2_ group (doublets, 1–1.8 ppm). After the oxidation, only one compound was found in the NMR spectrum, which could be assigned to **19** (Fig. 3c). While the two protons of the remaining double bond are split into two, more distinguished signals at 4.9 and 5.4 ppm, the signals corresponding to the bridgehead atoms experience a strong upfield shift (to 4.6, 3.7 and 3.2 ppm).

## Conclusion

The reaction rates for different disubstituted norbornenes have been determined which spanned two orders of magnitude. While reduced reaction rates were observed for all substituted norbornenes compared to unsubstituted norbornene, this was especially pronounced when electron-withdrawing substituents were introduced. For example, for (±)-*endo*,*exo*-dimethyl-5-norbornene-2,3-dicarboxylate, a very small reaction rate constant resembling those of unstrained alkenes was observed, while unsubstituted norbornene had a 100-fold higher reaction rate constant compared to styrene or hexene. Interestingly, when bearing electron-donating substituents, 2,3-*exo*-disubstituted norbornenes are reacting almost as fast as monosubstituted norbornenes which makes them interesting candidates for the preparation of multiple substituted iEDDA conjugation products. Overall, the following order of reactivity can be concluded: *Unsubstituted* > *exo* > *endo* > *exo*,*exo* > *endo*,*exo* > *endo*,*endo.*

## Figures and Tables

**Table 1:** Second order reaction rate constants for iEDDA reactions of different alkenes with pyTz

Compound		iEDDA second order rate constant [M^−1^ s^−1^]
**1**		0.155 ± 0.002
**2**^a^		0.11 ± 0.004
**3**		0.087 ± 0.009
**4**		0.073 ± 0.005
**5**		0.011 ± 0.0004
**6**		0.020 ± 0.0001
**7**		0.008 ± 0.001
**8**		0.012 ± 0.001
**9**		0.005 ± 0.0001
**10**		0.002 ± 0.0003
**11**		0.001 ± 0.0001
**12**		0.024 ± 0.0005
**13**		0.041 ± 0.002
**14**^b^		0.10 ± 0.002
**15**		0.008 ± 0.0003
**16**		0.001 ± 0.0002
**17**		0.003 ± 0.0003

a 80/20 *endo*-/*exo*-form.

b 95/5 *endo*-/*exo*-form.
